# VILO SLAM: Tightly Coupled Binocular Vision–Inertia SLAM Combined with LiDAR

**DOI:** 10.3390/s23104588

**Published:** 2023-05-09

**Authors:** Gang Peng, Yicheng Zhou, Lu Hu, Li Xiao, Zhigang Sun, Zhangang Wu, Xukang Zhu

**Affiliations:** 1 School of Artificial Intelligence and Automation, Huazhong University of Science and Technology, Wuhan 430074, China; 2 Key Laboratory of Image Processing and Intelligent Control, Ministry of Education, Wuhan 430074, China; 3 Shantui Construction Machinery Co., Ltd., Jining 272073, China

**Keywords:** multi-sensor fusion, pose estimation, lidar, visual inertial system

## Abstract

For the existing visual–inertial SLAM algorithm, when the robot is moving at a constant speed or purely rotating and encounters scenes with insufficient visual features, problems of low accuracy and poor robustness arise. Aiming to solve the problems of low accuracy and robustness of the visual inertial SLAM algorithm, a tightly coupled vision-IMU-2D lidar odometry (VILO) algorithm is proposed. Firstly, low-cost 2D lidar observations and visual–inertial observations are fused in a tightly coupled manner. Secondly, the low-cost 2D lidar odometry model is used to derive the Jacobian matrix of the lidar residual with respect to the state variable to be estimated, and the residual constraint equation of the vision-IMU-2D lidar is constructed. Thirdly, the nonlinear solution method is used to obtain the optimal robot pose, which solves the problem of how to fuse 2D lidar observations with visual–inertial information in a tightly coupled manner. The results show that the algorithm still has reliable pose-estimation accuracy and robustness in many special environments, and the position error and yaw angle error are greatly reduced. Our research improves the accuracy and robustness of the multi-sensor fusion SLAM algorithm.

## 1. Introduction

There are many excellent visual simultaneous localization and mapping (SLAM) systems, such as LSD-SLAM [1], DSO [2], and SVO [3]. These algorithms are based on pure vision. When there are no visual features, the accuracy and robustness of the pose estimation decreases rapidly, and the algorithm may fail. Therefore, in the follow-up development of visual SLAM, in order to overcome the shortcomings of pure vision, the strategy of multi-sensor fusion is adopted.

Because the inertial measurement unit (IMU) sensor-based inertial navigation algorithm has better short-term tracking performance, it can provide short-term accurate and reliable poses in the case of loss of visual features (light changes, missing texture features, and fast motion). In addition, the pure visual SLAM algorithm can provide speed constraints for the IMU-based inertial navigation algorithm, which can avoid the rapid spread of IMU measurement speed errors to a certain extent. Therefore, researchers have adopted the idea of loosely coupled fusion and used filters to fuse vision and IMU information to construct visual inertial SLAM algorithms. For example, the visual inertial SLAM algorithm in the literature [4,5,6,7] regards the IMU-based inertial navigation algorithm as the state prediction equation of the extended Kalman filter (EKF), and it uses the result of pure visual pose estimation as the measurement update equation of the EKF to realize the loosely coupled fusion of vision and IMU. Loosely coupled fusion means that the IMU and the camera estimate motion separately and then fuse their pose estimation results. In the loosely coupled fusion idea, because the visual features are invisible in the state optimizer, they cannot be adjusted by the information of the IMU. When encountering the positions of visual feature points with poor accuracy, the accuracy of the previous pure visual estimation decreases, and finally, the accuracy of the estimation of the whole state optimizer decreases. Tightly coupled fusion optimizes the camera pose and the position of the visual feature point together as the optimization variable of the entire state optimizer, which can optimize the position of the visual feature while optimizing the pose. This fusion strategy can effectively improve the disadvantages of loose coupling.

Although the addition of the IMU improves the accuracy and robustness of pure visual SLAM algorithms, the performance of visual–inertial SLAM systems can still be adversely affected by issues such as low lighting conditions, varying depth of field, and occlusion in complex environments, regardless of tightly coupled fusion or loosely coupled fusion. Moreover, due to the limitations of IMU precision, visual–inertial systems often struggle to maintain stability during long-term operation in weakly textured and unstructured scenes. Therefore, it is necessary to add high-precision ranging sensors, such as lidar. Lidar has the characteristics of strong anti-interference and high precision. When the visual features are lost, the lidar odometer can provide speed constraints for the IMU odometer and suppress the rapid spread of IMU measurement errors. At the same time, the motion distortion of lidar data can also be effectively corrected by the IMU, thereby improving the accuracy and robustness of the SLAM system.

In this paper, we propose a tightly coupled visual–inertial–lidar odometry framework, called VILO SLAM, by incorporating a low-cost 2D lidar into a visual–inertial SLAM system, to improve the positioning accuracy and robustness in complex environments. We combine the advantages of vision, IMU, and low-cost 2D lidar to make up for the insufficient information of each and build a tightly coupled state estimation problem based on nonlinear optimization. The main contributions of our work can be summarized as follows:

- Tightly coupling low-cost 2D lidar observations with stereo vision and inertial observations improves the accuracy and robustness of pose estimation in traditional visual–inertial SLAM algorithms in scenarios where visual features are lost due to darkness, strong light, or lack of texture.
- A lidar residual factor is constructed using the 2D lidar odometry model, and the Jacobian matrix of lidar residuals with respect to the state variables to be estimated is derived.
- The residual constraint equation of vision-IMU-LiDAR is constructed, and optimal robot pose estimation is obtained using nonlinear optimization, which solves the problem of fusing 2D lidar observations with binocular visual–inertial information in a tightly coupled manner.

## 2. Related Work

The most commonly used strategy in visual–inertial odometry is the tightly coupled approach, where vision and inertial measurements are combined into the same state vector. This method leverages visual measurements (such as feature points) and inertial measurements (such as accelerations and angular velocities) to construct error terms that include residuals from both sources. By optimizing the visual state variables and inertial measurement state variables simultaneously, the system achieves tightly coupled estimation. For example, Leutenegger et al. used a tightly coupled strategy for IMU measurement and integrated it into the key frame-based beam adjustment optimization visual SLAM. They then designed a tightly coupled SLAM algorithm called OKVIS based on a monocular camera and IMU [8]. In the SLAM process of this algorithm, there is a problem of repeated calculation of IMU points, which reduces the real-time performance of the entire algorithm. To solve this problem, the Christian Forster team designed the IMU pre-integration algorithm [9] and tightly coupled it with visual feature points to realize the visual inertial SLAM algorithm. Campos et al. fused IMU pre-integration and visual measurement to launch the ORB-SLAM3 system [10]. Xiao et al. designed tightly coupled real-time visual–inertial odometry based on the sliding-window method [11]. Yuan et al. proposed a multi-sensor fusion-state estimator based on a feature point optical-flow-tracking monocular vision, IMU, and wheel odometer measurement [12]. Hashim and Eltoukhy utilized available measurements obtained from group velocity vectors, feature measurements, and an inertial measurement unit, and proposed a computationally cheap geometric nonlinear SLAM filter algorithm that could account for the unknown bias inevitably present in velocity measurements [13]. Qin’s and Li’s teams at the Hong Kong University of Science and Technology adopted a sliding-window mechanism to construct the constraint equations of the IMU pre-integration error and the visual reprojection error. Additionally, they used a non-linear optimization method to propose the VINS-Fusion [14] SLAM algorithm, compatible with both monocular and binocular vision inertia. The algorithm also supports the expansion of fusion global sensor GPS to further improve the accuracy and robustness of the algorithm.

When visual features are lost, the visual–inertial SLAM algorithm degenerates into a classic inertial navigation algorithm. If the visual features cannot be recovered for a long time and the robot is in a state of uniform motion, resulting in no excitation of the IMU, the pose deviation estimated by the inertial navigation algorithm increases rapidly over time. Therefore, some researchers merge lidar, which has a strong anti-interference ability, with vision or IMU to further improve the accuracy and robustness of the visual SLAM algorithm. For example, in [15], Tixiao Shan constructed the residual constraint expressions of LiDAR and IMU pre-integration, computed robot poses through factor graph optimization, and proposed the LIO-SAM algorithm. In [16], a laser–inertial–vision tightly coupled SLAM framework is constructed, which improves the accuracy of robot pose estimation. In [17], the EKF is used to achieve loosely coupled fusion between the pure binocular vision-based pose-estimation results and the 2D lidar-based pose-estimation results, and a loosely coupled SLAM algorithm based on binocular vision-2D lidar is designed. However, the loose coupling of 2D lidar information and visual information has the problem of accuracy in specific scenes. As with the previously discussed visual-IMU loosely coupled fusion, if the position accuracy of the visual feature points is low, the accuracy of the entire fusion algorithm decreases.

Thus far, for unknown and complex indoor and outdoor environments, there are few studies on SLAM technology based on vision, inertia, and 2D lidar for tightly coupled optimization of multi-sensor fusion, and there is no mature solution similar to VINS-Fusion and ORB-SLAM.

## 3. Multi-Sensor Pose Estimation Based on Tightly Coupled Optimization

**Figure 1:**
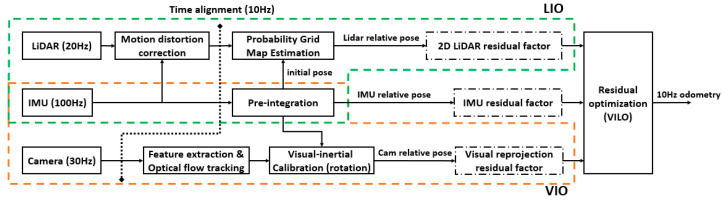
The system structure of VILO.

An overview of the proposed system is shown in Figure 1. The system receives sensor data from a 2D LiDAR, IMU, and a stereo camera. We seek to estimate the robot’s state and trajectory using these observations. The state estimation problem can be formulated as a maximum a posteriori (MAP) problem. Under the assumption of a Gaussian noise model, solving this MAP inference is equivalent to solving a nonlinear least-squares problem. In the system, we construct residual constraints of the 2D LiDAR, IMU, and camera, and perform optimization using Google’s Ceres nonlinear optimization library. Finally, the system outputs the pose of the robot in the world coordinate system at 10 Hz.

**Figure 2:**
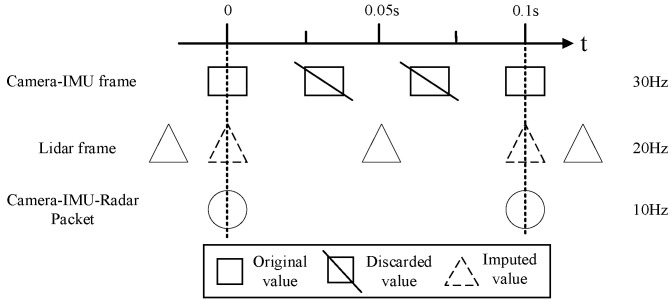
Camera-lidar time alignment diagram.

Due to the large amount of camera data, the camera-IMU frame should be reduced to 10 Hz considering the real-time performance of the robot pose solution. In addition, different sensor data acquisition frequencies are not consistent. If the time synchronization between sensors is not correct, the data provided by them may not be collected at the same time, which may lead to certain noise between data and affect the accuracy and robustness of the system. Therefore, the camera, IMU, and lidar need to be aligned in time, as shown in Figure 2.

First, the camera frame is used as the alignment mark, and the lidar data frame is interpolated at the 10 Hz time position of the camera-IMU frame. The interpolation of lidar data frames needs to be completed in combination with IMU. The specific operation can be divided into two steps. The first step is to record the time stamp of the nearest lidar frame before the camera-IMU frame timestamp and to calculate the time difference between the two. The second step is to integrate the original IMU data according to the time difference, calculate the pose change and transform the lidar data frame to the camera-IMU frame time. Then the data of the camera-IMU-lidar are collected into a data structure for subsequent processing.

The multi-sensor tightly coupled pose estimation method in this section is designed based on the binocular-IMU local pose estimator in the VINS-Fusion algorithm. Its innovation lies in the fusion of 2D lidar information with binocular vision-IMU pose estimation through tight coupling to form the binocular vision-IMU-2D lidar odometry (VILO) front-end pose estimator. It also lies in the addition of lidar observations to further restrict the pose to improve the accuracy and robustness of the SLAM front-end pose estimation in indoor and outdoor environments. The state variable χ to be solved in VILO is shown in the following formula:

(1) $$\begin{array}{l} \chi_{w}=\left[x_{0},x_{1},\cdots x_{n},\lambda_{0},\lambda_{1},\cdots \lambda_{m}\right]\\ x_{k}=\left[P_{b_{k}}^{w},R_{b_{k}}^{w},v_{b_{k}}^{w},b_{a_{k}},b_{g_{k}}\right],k\in [0,n] \end{array}$$

w represents the world coordinate system. In the state variable χ, $x_{k}$ is the IMU state when the *k*-th image is captured. It contains the position, speed, and direction of the IMU in the world coordinate system, as well as the accelerometer bias and gyroscope bias in the IMU body coordinate system. Additionally, *n* is the total number of key frames, *m* is the total number of feature points in the sliding window, and $\lambda_{i}$ is the inverse depth of the *i*-th observed feature point.

In VILO’s local pose estimation process, the observation set used to constrain the variable to be estimated is defined by the following formula:

(2) $$Z=\left[Z_{c_{i}},b_{i,i+1},L_{i,i+1}\right]\begin{array}{cc} & \left(i,i+1\right) \end{array}\in \kappa$$

In the formula, $Z_{c_{i}}$ represents the visual measurement, $b_{i,i+1}$ is the body coordinate system when the *i*-th and *i*+1-th images are acquired, and $L_{i,i+1}$ represents the measured value of the lidar odometry from the *i*-th key frame to the *i*+1-th key frame. κ is the key frame set in the sliding window.

**Figure 3:**
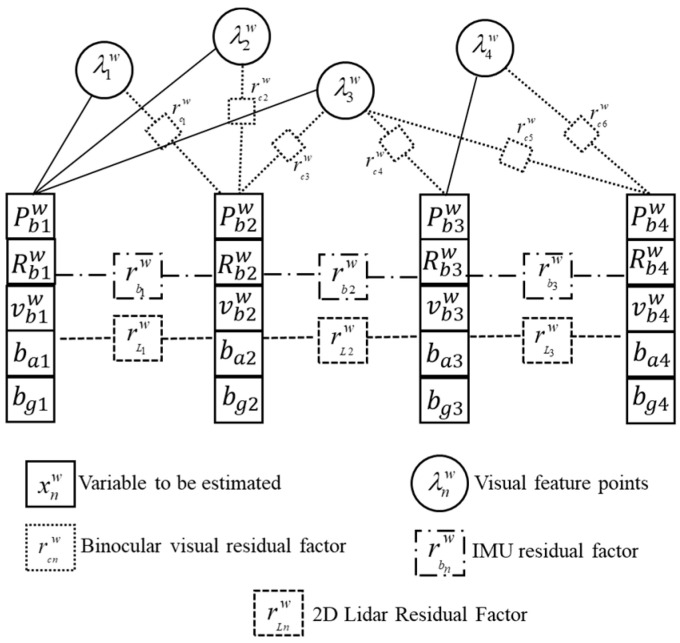
Binocular vision-IMU-2D lidar pose-estimation factor graph.

A factor graph (Figure 3) is constructed using the residual items in VILO as constraint factors and the variables to be optimized as nodes. In Figure 3, the circled node represents the inverse depth of the visual feature point observed by the camera, and the non-solid rectangular boxes are the visual residual factor, IMU residual factor, and lidar residual factor respectively. The variables to be estimated are restricted by the three residual factors. The construction of the IMU residual factor and the 2D lidar residual factor is only related to adjacent frames. The construction of the visual residual factor depends on the common-view relationship of feature points on adjacent frames. If the number of common-view feature points is scarce, the visual residual factor is not successfully constructed.

### 3.1. Nonlinear Least-Squares Model Based on Vision-IMU-Lidar

According to the residual factor of vision-IMU-lidar, and using the Mahalanobis distance to indicate the degree of deviation between the residual of each sensor and the covariance matrix, the VILO nonlinear least squares problem can be constructed as follows:

(3) $$\begin{array}{ll} {\chi_{w}}^{*}=\underset{\chi_{w}}{\arg\min} & \left({\left\|r_{p}-H_{p}\chi \right\|}^{2}+\sum_{(i,j)\in \kappa }{\left\|r_{b}({\left\{{\hat{a}}_{t},{\hat{\omega }}_{t}\right\}}_{t_{i}\leq t\leq t_{j}},x_{i},x_{j})\right\|}_{\Sigma_{b_{i,j}}}^{2}\right)\\ & +\sum_{i\in \kappa }\sum_{m\in \gamma_{i}}\rho ({\left\|r_{c}({\hat{z}}_{i}^{m},x_{i})\right\|}_{\Sigma_{c_{i,m}}}^{2})\\ & +\sum_{(i,j)\in \kappa }\rho \left({\left\|r_{L}(z_{t}^{L}-h_{t}^{L}{\left(\chi \right)}_{t_{i}\leq t\leq t_{i+1}},x_{i},x_{i+1})\right\|}_{\Sigma_{b_{i,i+1}}}^{2}\right) \end{array}$$

In (3), ${\left\|r\right\|}_{\Sigma }^{2}$ is the Mahalanobis distance of the residual *r* when the covariance matrix is Σ. The Mahalanobis distance is defined as ${\left\|r\right\|}_{\Sigma }^{2}=r^{T}\Sigma^{-1}r$. The first term is the prior information of the marginalization of the key frames in the sliding window, $r_{b}$ is the residual item of the IMU, $r_{c}$ is the binocular vision residual item, $r_{L}$ is the residual error of the 2D lidar odometry, and κ is the key frame set in the sliding window. In order to improve the robustness, the Huber loss function ρ [18] is used for the visual residual $r_{c}$ and the 2D laser odometry residual $r_{L}$, where ρ is

(4) $$\rho \left(s\right)=\left\{\begin{array}{c} 1\;s\geq 1\\ 2\sqrt{s}-1\;s<1 \end{array}\right.$$

#### 3.1.1. Visual Reprojection Residual Constraint

The pose of the key frame is defined as the pose $\left(P_{b}^{w},R_{b}^{w}\right)$ of the IMU coordinate system b, relative to the world coordinate system w. A certain visual feature point k in binocular vision is observed for the first time in key frame i, and its 3D space position coordinate is a function of the pose $\left(P_{b_{i}}^{w},R_{b_{i}}^{w}\right)$ of the current key frame and the inverse depth value $\lambda_{k}$ of the feature point. Next, the feature point k will continue to be tracked in the subsequent key frame j. Then, we can formulate the visual reprojection residual representation of the feature point k, which is a function of the key frame *i* and *j* poses $\left(P_{b_{i}}^{w},R_{b_{i}}^{w}\right)$ and $\left(P_{b_{j}}^{w},R_{b_{j}}^{w}\right)$, and the inverse depth $\lambda_{k}$ of the feature point, defined as the following formula:

(5) $$r_{c}(z_{j}^{k},\chi )=r_{c_{jk}}(P_{b_{i}}^{w},R_{b_{i}}^{w},P_{b_{j}}^{w},R_{b_{j}}^{w},\lambda_{k})$$

The advantage of defining the visual reprojection error to the unit ball is that the algorithm can support a wide-angle-lens model with severe distortion. It is also applicable to general camera models. Any pixel in the camera can be mapped to a ray on the unit ball. Therefore, the visual residual $r_{c}$ based on the unit ball is:

(6) $$\begin{array}{l} r_{c_{jk}}(P_{b_{i}}^{w},R_{b_{i}}^{w},P_{b_{j}}^{w},R_{b_{j}}^{w},\lambda_{k})={\left[\begin{array}{cc} b_{1} & b_{2} \end{array}\right]}^{T}\cdot \left({\hat{P}}_{c_{j}}^{k}-\frac{P_{c_{j}}^{k}}{\left\|P_{c_{j}}^{k}\right\|}\right)\\ {\hat{P}}_{c_{j}}^{k}=K_{c}^{-1}({\hat{z}}_{j}^{k})=K_{c}^{-1}\left(\begin{array}{c} {\hat{u}}_{c_{j}}^{k}\\ {\hat{v}}_{c_{j}}^{k} \end{array}\right)\\ P_{c_{j}}^{k}=R_{b}^{c}(R_{w}^{b_{j}}(R_{b_{i}}^{w}(R_{c}^{b}\frac{1}{\lambda_{k}}K_{c}^{-1}\left(\begin{array}{c} {\hat{u}}_{c_{i}}^{k}\\ {\hat{v}}_{c_{i}}^{k} \end{array}\right)+P_{c}^{b})+P_{b_{i}}^{w})+P_{w}^{b_{j}})+P_{b}^{c} \end{array}$$

In (6), $K_{c}^{-1}$ is the inverse transformation of the camera projection matrix used to convert 2D pixel coordinates to 3D coordinates in the camera coordinate system; ${\hat{P}}_{c_{j}}^{k}$ represents the 3D space coordinates of the *k*-th feature point in the key frame j camera coordinate system, and is the observation value; and $P_{c_{j}}^{k}$ represents the 3D space coordinates of the *k*-th feature point in the key frame j camera coordinate system, and is the prediction value, which is transformed from key frame *i* to *j* through the pose $\left(P_{b_{i}}^{w},R_{b_{i}}^{w}\right)$ of the *i*-th key frame and the pose $\left(P_{b_{j}}^{w},R_{b_{j}}^{w}\right)$ of the *j*-th key frame. $\left[\begin{array}{cc} b_{1} & b_{2} \end{array}\right]$ represents the two orthogonal basis vectors of the tangent plane of the unit sphere, used to map the 3D camera coordinate difference to the unit sphere; and $\left(P_{b}^{c},R_{b}^{c}\right)$ is the pose from the IMU to the camera, obtained through pre-calibration.

#### 3.1.2. IMU Residual Constraints

The IMU sensor can measure the acceleration and angular velocity information of the robot. The amount of measurement information is small, and the measurement frequency of IMU is high. Therefore, when the initial pose of the robot is known, the IMU can be used to continuously integrate time to obtain the speed and pose of the robot relative to the reference coordinate in real time. However, the IMU measurement carries a lot of noise, or the robot moves at a constant speed. If only IMU is used to estimate the pose, the accuracy and reliability of the pose estimation are low. Therefore, the integral information from the IMU can be used for data complementary fusion with the information of sensors, such as vision sensors, to improve the accuracy and robustness of the pose estimation.

In the visual inertial odometry (VIO), the IMU integration information between key frames is added as a residual constraint to the entire BA (bundle adjustment) optimization. According to the IMU pre-integration model, the IMU residual is as follows:

(7) $$\begin{array}{ll} r_{b}(z_{b_{i+1}}^{b_{i}},\chi ) & =r_{b}({\left\{{\hat{a}}_{t},{\hat{\omega }}_{t}\right\}}_{t_{i}\leq t\leq t_{i+1}},x_{i},x_{i+1})\\ & =\left[\begin{array}{c} \delta \alpha_{b_{i+1}}^{b_{i}}\\ \delta \beta_{b_{i+1}}^{b_{i}}\\ \delta \theta_{b_{i+1}}^{b_{i}}\\ \delta b_{a}{}_{b_{i+1}}^{b_{i}}\\ \delta b_{g}{}_{b_{i+1}}^{b_{i}} \end{array}\right]=\left[\begin{array}{c} R_{w}^{b_{i}}(P_{b_{i+1}}^{w}-P_{b_{i}}^{w}+\frac{1}{2}g^{w}\Delta t_{i}^{2}-v_{b_{i}}^{w}\Delta t_{i})-{\hat{\alpha }}_{b_{i+1}}^{b_{i}}\\ R_{w}^{b_{i}}(v_{b_{i+1}}^{w}+g^{w}\Delta t_{k}-v_{b}^{w})-{\hat{\beta }}_{b_{i+1}}^{b_{i}}\\ 2{\left[{\left({\hat{\gamma }}_{b_{i+1}}^{b_{i}}\right)}^{-1}⊗R_{b_{i}}^{w}{}^{-1}⊗R_{b_{i+1}}^{w}\right]}_{xyz}\\ b_{ab_{i+1}}-b_{ab_{i}}\\ b_{gb_{i+1}}-b_{gb_{i}} \end{array}\right] \end{array}$$

Equation (7) subtracts the measured value of the IMU pre-integration from the predicted value to obtain the IMU predicted score residual item. Among the variables in the equation, $\delta \alpha_{b_{i+1}}^{b_{i}}$ is the pre-integrated three-dimensional coordinate position residual of the IMU, $\delta \beta_{b_{i+1}}^{b_{i}}$ is the pre-integrated velocity residual of the IMU, and $\delta \theta_{b_{i+1}}^{b_{i}}$ is the IMU pre-integrated pose residual, that is, the rotation error of the IMU three axes. $\delta b_{a}{}_{b_{i+1}}^{b_{i}}$ and $\delta b_{g}{}_{b_{i+1}}^{b_{i}}$ are the zero-point error residual items of the accelerometer and gyroscope, respectively, in the IMU.

##### 3.2. 2D Lidar Residual Error Constraints

Usually, 2D lidar is used in the field of ground mobile robots. The lidar information is passed through the Bayesian conditional probability $p(z_{t}|x_{t},m)$ model of the radar to establish the maximum likelihood estimation problem. Then, the maximum likelihood estimation is converted into a scanning matching problem based on a laser point cloud, and a nonlinear least-squares problem that minimizes the error of scanning matching probability is constructed. Finally, a nonlinear optimization method is used to solve the pose of the lidar relative to the reference coordinate system, which is called the 2D lidar odometry algorithm. Because the pose estimated by the odometry algorithm has the advantage of a continuous and accurate scale, it can be tightly coupled and fused with the pose estimated by the VIO algorithm to improve the accuracy and robustness of the visual SLAM algorithm in indoor and outdoor scenes.

###### 3.2.1. 2D lidar Odometry Algorithm

On the basis of obtaining lidar data, the pose-estimation problem of lidar in the map can be transformed into a nonlinear least-squares problem as shown in (8) [19]:

(8) $$\xi^{*}=\underset{\xi }{\arg\min}{\left[1-M(S_{i}(\xi ))\right]}^{2}$$

ξ is the pose of lidar relative to grid map coordinate system. $S_{i}(\xi )$ represents the coordinates of the end point of the *i*-th scanning ray of the laser in the grid map coordinate system when lidar is in the pose variable ξ. $M(S_{i}(\xi ))$ is the probability that the grid map is an obstacle at the given coordinate position $S_{i}(\xi )$. The purpose of (8) is to find the ξ variable that minimizes the objective function ${\left[1-M(S_{i}(\xi ))\right]}^{2}$. $S_{i}(\xi )$ is represented as follows:

(9) $$S_{i}\left(\xi \right)=\left(\begin{array}{cc} \cos\left(\theta \right) & -\sin\left(\theta \right)\\ \sin\left(\theta \right) & \cos\left(\theta \right) \end{array}\right)\left(\begin{array}{c} s_{i}{}_{,x}\\ s_{i,y} \end{array}\right)+\left(\begin{array}{c} p_{x}\\ p_{y} \end{array}\right)$$

In (9), $\xi =(p_{x},p_{y},\theta {)}^{T}$, where $p_{x}$ and $p_{y}$ represent the coordinates of the origin of the laser sensor coordinate system relative to the reference coordinate system, and θ is the heading angle of the robot. The purpose of (9) is to transform the observation point of lidar to the grid map coordinate system.

Therefore, the core of the scan matching algorithm is that at the beginning it gives an estimated value ξ of lidar with a smaller deviation from the actual pose, and then finds a pose increment Δξ according to the laser data acquired at the current time t so that the end point of the laser scan and the probability grid map are optimally matched at the current time t, that is, (10) is established:

(10) $$\Delta \xi^{*}\underset{\Delta \xi }{\arg\min}{\sum_{i=1}^{n}\left[1-M\left(S_{i}\left(\xi +\Delta \xi \right)\right)\right]}^{2}\to 0$$

In order to solve the optimal increment Δξ that minimizes (10) at the current time t, the Gauss–Newton iteration method is used to perform the first-order Taylor expansion on the nonlinear function $M\left(S_{i}\left(\xi +\Delta \xi \right)\right)$ to obtain (11):

(11) $$\Delta \xi^{*}\underset{\Delta \xi }{\arg\min}{\sum_{i=1}^{n}\left[1-M\left(S_{i}\left(\xi \right)\right)-\nabla M\left(S_{i}\left(\xi \right)\right)\frac{\partial_{S_{i}\left(\xi \right)}}{\partial_{\xi }}\Delta \xi \right]}^{2}\to 0$$

In (11), *i* is the *i*-th laser beam of the laser sensor at time *t*. Sum the *n* beams of laser light, and obtain the partial derivative of Δξ from the objective function in (11) so that the partial derivative is 0. Finally, the pose increment Δξ is as follows:

(12) $$\Delta \xi =H^{-1}{\sum_{i=1}^{n}\left[\nabla M\left(S_{i}\left(\xi \right)\right)\frac{\partial S_{i}\left(\xi \right)}{\partial \xi }\right]}^{T}\left[1-M\left(S_{i}\left(\xi \right)\right)\right]$$

In (12), $M\left(S_{i}\left(\xi \right)\right)$ represents the probability value of an obstacle at the coordinate $S_{i}\left(\xi \right)=(x_{i},y_{i})$. According to equation (9), $\left(x_{i},y_{i}\right)$ may not be an integer pair. To express $\nabla M\left(S_{i}\left(\xi \right)\right)$, assume that $P_{i,i}=(x_{i},y_{i})$, $P_{01}=(x_{0},y_{1})=(floor(x_{i}),Ceiling(y_{i}))$, then we can write the approximation of $\nabla M\left(S_{i}\left(\xi \right)\right)$, as shown in (13). The Hessian matrix *H* is as shown in (14), and the partial derivative of $S_{i}\left(\xi \right)$ to ξ is as shown in (15):

(13) $$\left\{\begin{array}{ll} \frac{\partial M\left(P_{i,i}\right)}{\partial x} & \approx \frac{y_{i}-y_{0}}{y_{1}-y_{0}}\left(M\left(P_{11}\right)-M\left(P_{01}\right)\right)\\ & +\frac{y_{1}-y_{i}}{y_{1}-y_{0}}\left(M\left(P_{10}\right)-M\left(P_{00}\right)\right)\\ \frac{\partial M\left(P_{i,i}\right)}{\partial y} & \approx \frac{x_{i}-x_{0}}{x_{1}-x_{0}}\left(M\left(P_{11}\right)-M\left(P_{10}\right)\right)\\ & +\frac{x_{1}-x_{i}}{x_{1}-x_{0}}\left(M\left(P_{01}\right)-M\left(P_{00}\right)\right) \end{array}\right.$$

(14) $$H=\sum_{i=1}^{n}{\left[\nabla M(S_{i}(\xi ))\frac{\partial S_{i}(\xi )}{\partial \xi }\right]}^{T}\left[\nabla M(S_{i}(\xi ))\frac{\partial S_{i}(\xi )}{\partial \xi }\right]$$

(15) $$\frac{\partial S_{i}(\xi )}{\partial \xi }=\left(\begin{array}{llll} 1 & 0 & -\sin(\theta )s_{i}{}_{,x} & -\cos(\theta )s_{i,y}\\ 0 & 1 & \cos(\theta )s_{i}{}_{,x} & -\sin(\theta )s_{i,y} \end{array}\right)$$

The core of the 2D lidar odometry algorithm is to use the first-order Taylor series expansion of the Gauss–Newton iteration method to approximately replace the nonlinear least squares problem. The objective function $1-M(S_{i}(\xi ))$ is approximated near the lidar pose ξ, and multiple optimization iterations are performed until the increment Δξ is small enough, at which time iteration is stopped, the optimal variable ξ at the current time t is obtained, and (11), the formula of the residual sum of squares of the original model, is minimized.

The above analysis shows that the operating speed of the 2D lidar odometry algorithm is quite fast. Using the incremental formula (12), only a few simple iterative calculations are required to obtain the laser pose $\xi_{t}=(p_{x},p_{y},\theta {)}^{T}$ at time t.

##### 3.2.2. Residual Items Based on 2D Lidar Odometry Model

The residual factor $r_{L}$ of the 2D lidar pose can be expressed by the relative displacement $\Delta {\hat{P}}_{L_{i+1}}^{L_{i}}$ between the *i*-th frame and the *i*+1-th frame observed by the lidar odometry, and the relative displacement $\Delta P_{L_{i+1}}^{L_{i}}$ of the last two key frames before and after the state variable to be optimized. $\Delta {\hat{P}}_{L_{i+1}}^{L_{i}}$ and $\Delta P_{L_{i+1}}^{L_{i}}$ are, respectively, called the observed value and the predicted value. The specific form is described in the following formula:

(16) $$r_{L}(z_{t}^{L}-h_{t}^{L}{\left(\chi \right)}_{t_{i}\leq t\leq t_{i+1}},x_{i},x_{i+1})=z_{t}^{L}-h_{t}^{L}(x_{i},x_{i+1})=\Delta {\hat{P}}_{L_{i+1}}^{L_{i}}-\Delta P_{L_{i+1}}^{L_{i}}$$

In (16), L represents the lidar coordinate system. $x_{i}=(P_{b_{i}}^{w},R_{b_{i}}^{w})$ represents the pose of the IMU relative to the world coordinate system at the *i*-th frame.$h_{t}^{L}{\left(\chi \right)}_{t_{i}\leq t\leq t_{i+1}}$ represents the relative pose estimation between the *i*-th frame and the *i*+1-th frame. The residual term does not include the error term about the pose but rather the error term about the position. The reason why the error term of the pose is discarded is that the IMU can provide a higher-precision integration of the pose angle. Therefore, pose is not used as a residual constraint here. The specific expansion form of $h_{t}^{L}(x_{t-1},x_{t})$ (that is, $\Delta P_{L_{i+1}}^{L_{i}}$) is shown in the following formula:

(17) $$\begin{array}{ll} h_{t}^{L}(x_{i},x_{i+1}) & =\Delta P_{L_{i+1}}^{L_{i}}=R_{w}^{L_{i}}(P_{L_{i+1}}^{w}-P_{L_{i}}^{w})\\ & =R_{w}^{L_{i}}(P_{b_{i+1}}^{w}+R_{b_{i+1}}^{w}P_{L}^{b}-P_{b_{i}}^{w}-R_{b_{i}}^{w}P_{L}^{b})\\ & =R_{w}^{L_{i}}(P_{b_{i+1}}^{w}-P_{b_{i}}^{w})+R_{w}^{L_{i}}R_{b_{i+1}}^{w}P_{L}^{b}-R_{w}^{L_{i}}R_{b_{i}}^{w}P_{L}^{b})\\ & =R_{b}^{L}R_{w}^{b_{i}}(P_{b_{i+1}}^{w}-P_{b_{i}}^{w})+R_{b}^{L}R_{w}^{b_{i}}R_{b_{i+1}}^{w}P_{L}^{b}-R_{b}^{L}P_{L}^{b} \end{array}$$

Finally the residual term of the relative displacement $\Delta {\hat{P}}_{L_{i+1}}^{L_{i}}$ between the position and pose of the variable to be optimized $x_{i}=(P_{b_{i}}^{w},R_{b_{i}}^{w})$, $x_{i+1}=(P_{b_{i+1}}^{w},R_{b_{i+1}}^{w})$ and the lidar odometry frames *i* and *i*+1 are obtained as follows:

(18) $$r_{L}=\Delta {\hat{P}}_{L_{i+1}}^{L_{i}}-R_{b}^{L}R_{w}^{b_{i}}(P_{b_{i+1}}^{w}-P_{b_{i}}^{w})-R_{b}^{L}R_{w}^{b_{i}}R_{b_{i+1}}^{w}P_{L}^{b}+R_{b}^{L}P_{L}^{b}$$

In (18), $\Delta P_{L_{i+1}}^{L_{i}}$ can be obtained by lidar odometry, and $\left(R_{L}^{b},P_{L}^{b}\right)$ is the pose of the lidar coordinate system relative to the IMU coordinate system, which can be obtained by pre-calibration.

Because the lidar odometry can only observe the pose yaw angle θ, the residual model can only constrain the pose yaw angle in the state variable χ to be optimized. Then the state variable χ is specifically $\chi =(P_{b_{i}}^{w},\theta_{b_{i}}^{w},P_{b_{i+1}}^{w},\theta_{b_{i+1}}^{w})$, and the Jacobian matrix of the residual term $r_{L}$ with respect to the state variable χ to be estimated is as follows:

(19) $$\begin{array}{l} J{\left[0\right]}^{3\times 10}=\left[\begin{array}{cc} \frac{\partial r_{L}}{\partial P_{b_{i}}^{w}} & \frac{\partial r_{L}}{\partial \theta_{b_{i}}^{w}} \end{array}\right]=\left[\begin{array}{cc} -R_{b}^{L}R_{w}^{b_{i}} & -R_{b}^{L}{\left[R_{w}^{b_{i}}(P_{b_{i+1}}^{w}-P_{b_{i}}^{w}+R_{w}^{b_{i+1}}P_{L}^{b})\right]}^{\wedge } \end{array}\right]\\ J{\left[1\right]}^{3\times 10}=\left[\begin{array}{cc} \frac{\partial r_{L}}{\partial P_{b_{i+1}}^{w}} & \frac{\partial r_{L}}{\partial \theta_{b_{i+1}}^{w}} \end{array}\right]=\left[\begin{array}{cc} R_{b}^{L}R_{w}^{b_{i}} & -R_{b}^{L}R_{w}^{b_{i}}R_{w}^{b_{i+1}}{\left(P_{L}^{b}\right)}^{\wedge } \end{array}\right] \end{array}$$

In (19), ∧ is the transformation of the vector to the antisymmetric matrix. $J{\left[0\right]}^{3\times 10}$ and $J{\left[1\right]}^{3\times 10}$ are the first-order partial derivatives of the lidar residual $r_{L}$ with respect to the pose $\left(P_{b_{i}}^{w},\theta_{b_{i}}^{w}\right)$ and $\left(P_{b_{i+1}}^{w},\theta_{b_{i+1}}^{w}\right)$ of the *i*-th and *i*+1-th key frame, also known as the Jacobian matrix.

The covariance matrix $\Sigma_{L}$ of the residual term of the lidar odometry is the covariance matrix of the pose estimation in the lidar odometry model. The covariance matrix can be obtained by the laser scanning matching algorithm.

Thus far, the least squares problem based on binocular-IMU-lidar has been constructed, and the visual residual $r_{c}$, IMU residual $r_{b}$, and 2D lidar odometry residual $r_{L}$ have been analyzed. Furthermore, the Jacobian matrix analytical formula of the residual $r_{L}$ of the lidar odometry with respect to the state variable $\chi_{w}$ to be estimated has been derived. Therefore, Google’s Ceres nonlinear optimization library can be used to solve the least squares model. After three sensor measurements are jointly constrained and continuous iterative optimization is carried out, the pose estimation can be solved with high accuracy and robustness in indoor and outdoor environments.

### 3.3. Pseudo-Code Description

The pseudo code for VILO residual calculation and optimization is shown in Algorithm 1. Firstly, the SLAM system and variables are initialized. Secondly, the data from the 2D lidar, IMU, and camera are received and processed in a loop. It is important to note that motion distortion correction is performed on the 2D lidar scan data using the IMU raw data before time-aligning all the sensor data. After obtaining the time-aligned data packets, the IMU pre-integration residual, visual reprojection residual, and 2D lidar odometry residual are calculated. Finally, the final pose is solved using the Ceres nonlinear optimization library.
**Algorithm 1** VILO Residual Calculation and Optimization**Input:** IMU data, camera images, and 2D LiDAR point cloud data**Output:**$\mathrm{The}\;\mathrm{pose}\;\mathrm{of}\;\mathrm{robot}\;x=\left[P_{b}^{w},R_{b}^{w}\right]$ in the world coordinate system
// Step 1. Initialize system and variables1:$x_{i}=\left[P_{b_{i}}^{w},R_{b_{i}}^{w},v_{b_{i}}^{w},b_{a_{i}},b_{g_{i}}\right]$ //Initialize state variables according to Equation (1);2:$Z=\left[Z_{c_{i}},b_{i,i+1},L_{i,i+1}\right]$ //Initialize observation set according to Equation (2);3:// Step 2. Receive data and perform pose estimation and optimization4:**while** received IMU, camera image and 2D LiDAR point cloud **do**5: Undistorted pcd = Motion distortion correction (imu data, point cloud)6: Z=sync_process(imudata,image,Undistortedpcd)//Time alignment7: 
if empty(Z) then
8:  
continue;
9: 
**end if**
10: $r_{b}=processIMU(Z,x_{i})$//IMU residual according to Equation (7)11: F=featureTracker(Z) //Extract features (pixel coordinates) and optical flow tracking12: $r_{c}=processImage(F,x_{i})$ //Visual reprojection residual according to Equations (5) and (6)13: $\Delta \xi =probabilityGridMap(Z,x_{i})$ //LiDAR pose estimation according to Equations (8)–(15)14: $r_{L}=processLiDAR(\Delta \xi ,x_{i})$ //Construct 2D lidar odometry residual according to Equation (18)15: $x_{i+1}=optimization(r_{b},r_{c},r_{L})$ //Ceres optimization, get output pose16:**end while**

## 4. Experiment

In order to verify the accuracy and effectiveness of the tightly coupled pose estimation algorithm based on binocular VILO proposed in this paper, some extreme experimental environments need to be selected, such as insufficient light or darkness, lack of texture characteristics (indoor white walls), and frequent movement of dynamic obstacles (people). Therefore, this section focuses on the corridor environment of the indoor experimental building with extreme conditions for algorithm comparison and verification experiments. In this paper, we compare the performance of VILO (ours), VINS Fusion and ORB-SLAM2.

**Figure 4:**
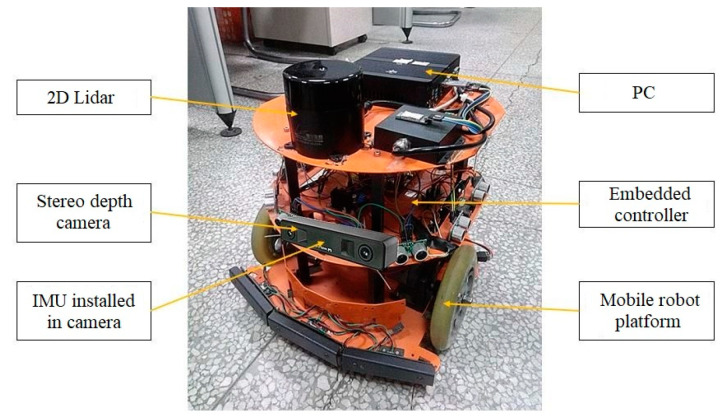
Sensor mounting platforms. PC is used for data acquisition of robot sensors, pose estimation and mapping. Embedded controller is used for robot motion control.

In this pose-estimation experiment of the three types of algorithms, it is necessary to use the cumulative error of each algorithm to measure the excellence of each algorithm. Therefore, the visual loop detection function is not turned on during the operation of the three types of algorithms because it would eliminate the cumulative pose error. All the methods are executed on a computing device equipped with an Intel i7-8700 CPU using the robot operating system (ROS) in Ubuntu Linux. The sensor mounting platforms are shown in Figure 4.

### 4.1. Motion Trajectory Comparison Experiment

**Figure 5:**
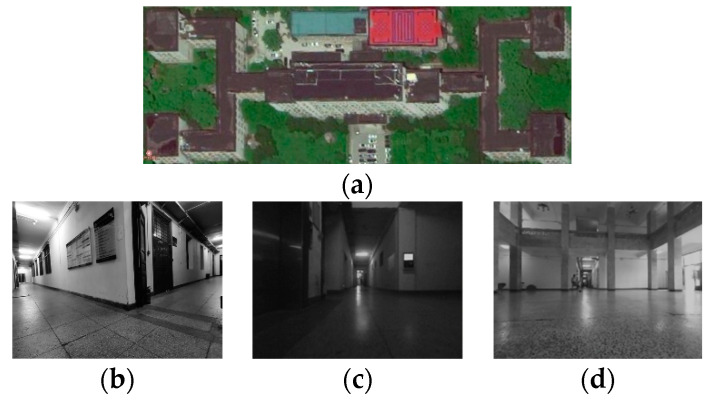
Indoor corridor environment. (**a**) Corridor outline; (**b**) Corner of the corridor; (**c**) Insufficient light inside the corridor; (**d**) Corridor hall scene.

Experimental configuration description: The algorithm verification environment is a corridor environment on the first floor of the experimental building with a width of 3 m, a long length, corners, and a relatively empty hall environment. The area on the first floor is about 250 × 100 m^2^. The scene of this experiment is shown in Figure 5, where (a) is the satellite map of the experimental building, in which you can clearly see the outline of the corridor, and (b–d) show the scene inside the corridor. The robot is controlled to traverse every scene in the corridor as much as possible, the linear velocity of movement is maintained at 0.5 m/s, and the angular velocity is maintained at 0.5 rad/s. A large number of fixed marking points are arranged inside the corridor to obtain the real position of the robot in order to analyze the positioning error of the robot.

The extreme situations in the experiment are as follows:

(1) Parts of the corridor walls are completely white without obvious visual features;
(2) In the corridor hall, the walls are covered with tiles with high reflectivity, which affects the camera observation data;
(3) The similarity of some corridor scenes is relatively high, and there are no special markings, which affects the accuracy of 2D lidar odometry;
(4) On some floors of the corridor, there are cracks, uneven heights, and large vibrations when the robot moves, causing large fluctuations in the measured values of each sensor;
(5) During the experiment, there were many pedestrians in the field of view of the camera and 2D lidar.

**Figure 6:**
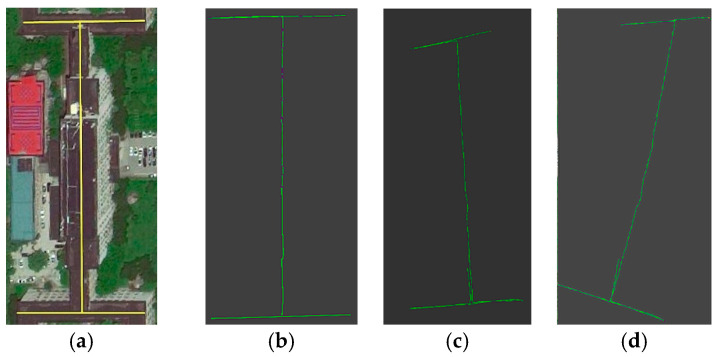
The trajectory of each algorithm (top view). (**a**) Standard trajectory; (**b**) This paper; (**c**) VINS-Fusion; (**d**) ORB-SLAM2.

The trajectories of each algorithm in an experiment are shown in Figure 6, including the standard trajectory of the corridor environment, the trajectory of the algorithm in this paper (binocular-IMU-2D lidar), the trajectory of the VINS-Fusion algorithm (binocular-IMU), and the trajectory of the ORB-SLAM2 algorithm (pure binocular vision).

The motion trajectory of the pose estimation algorithm in this paper, shown in Figure 6b, is highly consistent with the real corridor contour, demonstrating that it effectively restores the real robot motion trajectory, and the overall error is small. The trajectory of the VINS-Fusion algorithm in Figure 6c does not closely match the contour of the corridor. In the scene where some visual features of the corridor are missing, the pose-estimation drifts, causing the recorded trajectory to curve or be lost when compared to the actual trajectory of the robot. Especially in the lower part of the trajectory in Figure 6c, when the robot returns, it encounters the loss of visual features, and the robot also moves at a constant speed, meaning the IMU is not stimulated, which causes the VINS-Fusion algorithm to fail, and the algorithm exits early. The ORB-SLAM2 algorithm in Figure 6d has the largest deviation from the real contour of the corridor and thus the worst performance among the three algorithms. The reason for this is that it only relies on a single visual sensor. In the case of few visual features or high visual noise (more pedestrians), the pose-estimation error increases rapidly, resulting in a large deviation between the estimated motion trajectory and the true trajectory. Like the VINS-Fusion algorithm, in the lower part of the trajectory, when the robot returns, it encounters complete loss of visual features, and the algorithm also fails.

Because the algorithm proposed in this paper integrates lidar information, it overcomes the problem of the original binocular vision-IMU algorithm being overly dependent on visual information. In the case of loss of visual features and lack of IMU excitation, reliable and high-precision pose estimation can still be achieved. Because the lidar measurement value has strong anti-interference ability and can adapt to many extreme environments, after vision is restored, the robot can still combine the lidar-estimated pose for joint optimization to obtain a more precise pose.

### 4.2. Pose Estimation Accuracy Verification Experiment

The robot was manually controlled to repeat two to three closed-loop motions along the corridor environment, referring to the robot moving from the same starting position and then returning to the starting position. For each closed-loop motion, it was ensured that the ending pose at the starting point was consistent with the starting pose (the position error was less than 0.1 cm, and the heading angle error was less than 5°). Furthermore, rosbag, the message recording tool in ROS, was used to record binocular vision, IMU, and 2D lidar data in the same bag file.

Later, the recorded data set was applied to the ORB-SLAM2 algorithm, the VINS-Fusion algorithm, and the binocular vision inertial pose estimation algorithm of tightly coupled fusion 2D lidar proposed in this paper to conduct a positioning-accuracy-comparison verification experiment. The relative pose between the start point and the end point of the motion obtained by each pose-estimation algorithm was used as the pose error to evaluate the effect of the pose estimation.

The robot in this paper was a mobile robot on the ground, so only the three-dimensional pose $\left(p_{x},p_{y},\theta \right)$ of the robot coordinate system relative to the global coordinate system, where $p_{x},p_{y}$ is the offset of the X and Y axes, and θ is the yaw angle of the robot, needed to be recorded during the experiment. In order to more clearly illustrate the effectiveness of the pose estimation algorithm proposed in this paper, three-pose estimation experiments were performed on each of the three algorithms. First, the initial pose-estimation value of each algorithm, that is, the pose of the robot’s starting point, was recorded. Starting from the same starting point, the robot moved along the same path and returned to the starting point again, at which time the pose of the robot was recorded. By calculating the average value of the three motions of each experimental parameter of each algorithm and comparing the average value with the initial pose, the pose-estimation error of each algorithm was obtained.

The results of this pose-estimation experiment are shown in Table 1. We computed the mean absolute error (MAE) for each experiment, which represents the average absolute difference between the predicted and true values and is presented in the table as the absolute value of the “Average” minus “Initial pose” parameter in units of meters. This parameter can measure the accuracy of the pose estimation. To demonstrate the accuracy differences between algorithms more clearly, we separately displayed the MAE errors of the X axes, Y axes, and yaw angle for each algorithm, which better measures the differences between the algorithms.

From the analysis in Table 1, it can be seen that the ORB-SLAM2 algorithm has a large error in the pose estimation during the entire closed-loop motion process. The position estimation deviations of the X axis and Y axis both exceed 5 m, and the yaw angle error is also large. The pose-estimation error of the VINS-Fusion algorithm is much lower than that the ORB-SLAM2 algorithm. Because the VINS-Fusion algorithm uses IMU to make up for the lack of visual information, it performs well if the robot does not move at a constant speed, but this is not in line with reality. In real situations, the robot often moves at a constant speed, so the IMU information is invalid, and the VINS-Fusion algorithm degenerates to a pure visual pose-estimation algorithm. In the case of loss of visual features or large interference (more pedestrians), the reliability of the VINS-Fusion algorithm performance decreases. In the method of this paper, the average pose error estimation is the smallest. The position estimation error drops below 4 m, and the yaw angle error is much smaller than that of the VINS-Fusion algorithm. This is because 2D lidar odometry compensates for the degradation and instability issues of VINS-Fusion. When a robot moving at a constant speed enters a feature-sparse scene, the visual-inertial odometry is severely affected, while lidar odometry works normally. By tightly coupling low-cost 2D lidar, camera, and IMU, the accuracy and robustness of the pose estimation are effectively improved.

Overall, in terms of accuracy, after the robot moves about 758.62 m, the algorithm in this paper reduces the Euclidean distance of the position error by 38.7% and the yaw angle error by 48.4% compared with ORB-SLAM2. Compared with VINS-Fusion, the Euclidean distance of the position error is reduced by 20.8%, and the yaw angle error is reduced by 18.1%. In terms of robustness, after many experiments, the algorithm in this paper has not failed in many extreme situations (visual occlusion, uniform motion, and more pedestrians), so the algorithm is robust. In terms of real-time performance, the algorithm in this paper can achieve 10 Hz, which can meet the needs of tasks that do not require high real-time performance. Therefore, the tightly coupled fusion VILO pose estimation method proposed in this paper can effectively improve the accuracy and robustness of the robot pose estimation and has strong feasibility. It can provide reliable pose estimation for other modules of visual SLAM, such as environment mapping.

### 4.3. Comparative Experiment on Dense Mapping of Spatial Environment Based on Three Types of Pose-Estimation Algorithms

In order to further highlight the performance of the tightly coupled fusion 2D lidar pose-estimation algorithm proposed in this paper in real space environment mapping, a 3D dense mapping algorithm based on super-pixel bins [20] is applied to ORB-SLAM2, VINS-Fusion, and the pose-estimation algorithm proposed in this paper to conduct 3D dense-mapping-quality comparison experiments.

**Figure 7:**
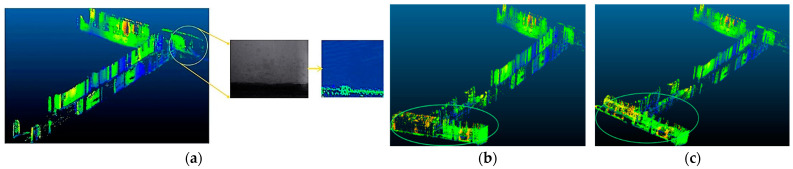
3D mapping comparison experiment based on three types of pose-estimation algorithms. (**a**) Based on ORB_SLAM2; (**b**) Based on VINS_Fusion; (**c**) Based on algorithm of this paper.

The 3D dense maps in Figure 7 are all represented by 3D dense point cloud maps. In Figure 7a, the middle image is an actual scene with a lack of visual features in a corridor, and the right image is a 3D dense point cloud image established at this scene, corresponding to the map position indicated by the circle in the left image. The loss of visual features causes the pose tracking of the ORB_SLAM2 algorithm to fail. As a result, the mapping algorithm fails to build the map at this location, the algorithm exits early, and ultimately the entire environment mapping is incomplete.

In Figure 7b, because the algorithm fuses binocular vision and IMU sensor information, when the visual features are lost, the algorithm can still perform short-term pose tracking based on IMU information. Therefore, compared with the mapping effect of ORB_SLAM2, based on this algorithm, the spatial 3D environment map can be completely established without visual loss. However, when the robot is moving at a constant speed, the acceleration of the IMU is not stimulated, which leads to a decrease in the short-term tracking accuracy of the algorithm and an increase in the cumulative error, which makes the established 3D map deform. The 3D dense map indicated by the ellipse in Figure 7b corresponds to a time when the robot was moving at a constant speed. The map shows that the lower part of the corridor has been deformed.

Figure 7c shows the dense 3D environment map established by the pose-estimation algorithm proposed in this paper. The map has no obvious distortion, indicating that the algorithm can ensure the accuracy and robustness of pose estimation under extreme conditions such as loss of visual features and uniform motion of the robot, and it truly restores the actual environment. According to the comparison of actual environment mapping experiments, the pose-estimation algorithm proposed in this paper can effectively overcome the robot-pose-estimation problem in some extreme environments and has good robustness.

## 5. Summary

Aiming to improve the accuracy and robustness of the visual–inertial SLAM algorithm, we designed and implemented VILO, which includes a binocular visual–inertial system and low-cost 2D lidar. With the help of 2D lidar odometry, the 3D pose of the 2D lidar relative to the local reference coordinate system is obtained before sensor data fusion. Then, a residual constraint equation can be constructed, which allows the low-cost 2D lidar information to be used to optimize with the binocular visual–inertial state estimator based on tightly coupled optimization, and the optimal robot pose is solved through nonlinear optimization. The experimental results show that the VILO SLAM algorithm can still have high pose accuracy and robustness in multiple extreme environments. We hope that our work can provide a feasible idea and scheme for SLAM of a mobile robot.

## Figures and Tables

**Table 1:** Corridor environment pose-estimation experiment results.

Algorithm	Parameters	Initial Pose	1st Finish Pose	2nd Finish Pose	3rd Finish Pose	Average	MAE
ORB-SLAM2	X axis offset (m)	0.032	6.56	5.36	7.28	6.40	6.368
	Y axis offset (m)	−0.028	−5.32	−5.98	−6.46	−5.92	5.892
	yaw angle (degrees)	0.351	8.563	7.253	10.26	8.692	8.341
VINS-Fusion	X axis offset (m)	−0.021	5.221	4.336	4.758	4.772	4.793
	Y axis offset (m)	0.033	−4.532	−5.142	−4.349	−4.674	4.707
	yaw angle (degrees)	0.283	5.286	5.463	5.852	5.534	5.251
Our method	X axis offset (m)	−0.011	−3.326	−3.635	−3.867	**−3.609**	**3.598**
	Y axis offset (m)	0.016	−3.855	−4.126	−3.732	**−3.904**	**3.920**
	yaw angle (degrees)	−0.203	4.563	4.068	3.659	**4.10**	**4.303**

## Data Availability

Data sharing not applicable.
